# Pulsed Light Treatment Effect on Color, Oxidative Stability, and *Listeria monocytogenes* Population of Sliced Mortadella

**DOI:** 10.3390/foods13182976

**Published:** 2024-09-19

**Authors:** Priscila Rossato Fracari, Ana Guimarães Massia, Denise Adamoli Laroque, Bibiana Alves dos Santos, Alexandre José Cichoski, Bruno Augusto Mattar Carciofi, Paulo Cezar Bastianello Campagnol

**Affiliations:** 1 Department of Technology and Food Science, Federal University of Santa Maria, Santa Maria 97105-900, RS, Brazil; priscila_434@hotmail.com (P.R.F.); bialvesantos@gmail.com (B.A.d.S.); cijoale@gmail.com (A.J.C.); 2 Department of Chemical and Food Engineering, Federal University of Santa Catarina, Florianópolis 88040-900, SC, Brazil; anagmassia@gmail.com (A.G.M.); laroquedenise@gmail.com (D.A.L.); carciofi@ucdavis.edu (B.A.M.C.); 3 Department of Biological and Agricultural Engineering, University of California Davis, Davis, CA 95616, USA

**Keywords:** non-thermal treatment, emulsified meat products, post-cooking contamination, lipid oxidation, sensory profile

## Abstract

This study evaluated the effects of high-intensity pulsed light (PL) on sliced mortadella, assessing how the parameters pulse width (1260 to 2520 µs) and number of pulses (one to three) influence color, oxidative stability, and *Listeria monocytogenes* population. The different PL parameters generated a fluence ranging from 2.64 to 6.57 J/cm^2^ and irradiance ranging from 1046.9 to 1738.8 W/cm^2^. The PL slightly increased the temperature and pH of the samples, and this elevation was well correlated to the higher number of pulses and higher fluence. The color parameter a* was reduced while b* values increased after PL application, with these effects being more significant in treatments with a higher number of pulses and higher fluence. The highest values of TBARS were found in treatments with higher fluence (5.28 and 6.57 J/cm^2^), which were characterized by the attribute “oxidized color” in sensory evaluation. The different PL conditions reduced the count of *L. monocytogenes* by up to 1.44 Log CFU/cm^2^. The treatment with a pulse width of 1260 µs, two pulses, fluence of 4.38 J/cm^2^, and irradiance of 1738.3 W/cm^2^ achieved the same efficacy in pathogen reduction as the treatments with higher fluence. Moreover, these PL conditions had a minimal impact on the color and oxidative stability of mortadella, demonstrating an effective balance between microbiological safety and quality preservation.

## 1. Introduction

Mortadella is a meat product widely consumed in various parts of the world and represents a significant percentage of the economic yield for many industries [1]. A considerable amount of mortadella is marketed as sliced and vacuum-packed. However, if any microbiological contamination occurs during the post-cooking stages, the shelf life and safety of the product will be drastically compromised.

Contamination by *L. monocytogenes* is a reality in many industries, and several outbreaks with fatal consequences have been reported worldwide [2,3,4]. In addition to this microorganism’s high pathogenicity, another factor of concern is its ability to multiply at refrigeration temperatures [5]. Moreover, mortadella is a ready-to-eat product, making it a potential agent for transmitting foodborne diseases if contamination occurs after the thermal treatment or if this treatment is not performed correctly [6]. Studies reported that even in industries with rigorous hygienic–sanitary practices, it is not easy to eliminate *L. monocytogenes* [7,8]. For this reason, in some countries, such as the United States, regulatory bodies impose strict guidelines for meat products that are exposed to the environment after cooking, such as sliced mortadella, requiring specific measures to control *L. monocytogenes* contamination at these stages [9]. This requirement highlights the meat industry’s challenge in ensuring the safety of ready-to-eat meat products that undergo post-cooking processes with environmental exposure. Since traditional heat sterilization is impractical for post-packaged ready-to-eat products due to the potential deterioration of sensory qualities such as texture, flavor, and appearance [10], non-thermal decontamination methods are preferred. Therefore, researching new strategies, especially environment-friendly and efficient ones, to combat this microorganism is paramount.

In this regard, high-intensity pulsed light (PL), a non-thermal surface decontamination technology [11], emerges as a potential alternative to address this problem faced by the sliced meat product industry. The United States Food and Drug Administration (FDA) approves this technology, offering advantages such as high efficiency and sustainability [12]. Moreover, studies have proven its efficacy in reducing the microbial contamination of various food products, such as *Escherichia coli* and *Enterococcus faecium* on the surface of shell eggs [13,14,15], *Listeria innocua* in sliced cheese [16], *Pseudomonas fluorescens*, *E. coli* ATCC 25922, and *L. innocua* on cheese surfaces [11], and *E. coli* O157:H7 in Romaine lettuce [15]. PL is an economically advantageous technology that uses inert gas lamps, typically xenon gas [17]. Its antimicrobial mechanism involves the transformation of high-power electrical pulses into broad-spectrum light that causes irreversible damage to the DNA of microorganisms due to the formation of thymine pyrimidine and cytosine dimers [18,19,20]. However, its use needs to be optimized for each type of food, as factors such as chemical composition, surface characteristics, and product thickness can influence the efficacy of PL [10,21].

Despite PL being effective in reducing microorganisms in foods, it can cause some alterations that compromise the quality of the product. One possible alteration is the increase in lipid oxidation due to the high energy applied to the food [22]. PL can also alter the color of foods [23], which is one attribute that primarily impacts consumers’ purchasing decisions, especially in the case of sliced meat products. Therefore, it is essential to determine the best conditions for applying PL that allow for increased microbiological safety while minimal adverse effects on the color and lipid oxidation of the food product take place [24].

A growing interest in PL application to foods is observed. Still, there is a gap in the scientific literature regarding PL-specific use in highly consumed ready-to-eat meat products, such as mortadella. In this context, the present study sought to investigate how the PL parameters pulse width and number of pulses impact the quality attributes of color and oxidative stability when aiming to reduce the *L. monocytogenes* population in sliced mortadella.

## 2. Materials and Methods

### 2.1. Production of Mortadellas

Mortadella was elaborated with the following formulation: beef (70%), pork backfat (15%), sodium chloride (NaCl) (2.5%), sodium tripolyphosphate (STPP) (0.5%), sodium nitrite (0.015%), sodium erythorbate (0.025%), monosodium glutamate (0.3%), garlic powder (0.1%), coriander powder (0.1%), black pepper (0.1%), and ice (11.36%). The beef and pork backfat were ground using a 5 mm disc (PJ-22 Plus Professional, Jamar, Tupã, Brazil). The beef was then placed in a cutter (Model 0.5L 60 Hz, G-Paniz, Francisco Beltrão, Brazil) and minced with NaCl and STPP for 30 s. After the addition of the other ingredients and additives, the mincing continued for another 30 s. Then, the pork backfat and ice were added, and the mincing was continued until a homogeneous mass was obtained. The mass temperature was kept below 12 °C throughout the mincing process. The meat mass was stuffed into plastic casings with a thickness of 46 µ and a diameter of 32 mm (Viscofan, São Paulo, Brazil), forming 150 g pieces. The cooking was carried out in water at 80 °C until an internal temperature of 72 °C was reached. The mortadellas were cooled in an ice bath for 20 min and stored at 4 °C for 24 h. The mortadella centesimal composition was determined based on the AOAC (2010) methodology, and the following values were obtained: 64.4 ± 1.2% moisture, 17.9 ± 0.5% lipids, and 16.1 ± 0.2% proteins [25].

Mortadella pieces were sliced to a thickness of 2.5 mm (approximately 1 g and area of 9.08 cm^2^) on an electric slicer (CFIF 275, Metvisa, Brusque, Brazil) previously sanitized with 70% alcohol. The samples were divided into two groups: PL on non-inoculated samples and PL on inoculated samples.

### 2.2. L. monocytogenes Suspension

A lyophilized strain of *L. monocytogenes* (CCT 7474) isolated from sausages from the André Tosello Foundation (Campinas, Brazil) was rehydrated and cultured in BHI broth (Brain Heart Infusion, Kasvi, Brazil) at 37 °C for 24 h. Subsequently, using a bacteriological loop, the rehydrated strain was streaked on the surface of Petri dishes containing ALOA agar (Agar Listeria Ottaviani & Agosti, Neogen, Lansing, MI, USA). The plates were incubated at 37 °C for 48 h. Afterward, a colony-forming unit from the ALOA agar plates was transferred to tubes containing 5 mL of TSB broth (Tryptone Soy Broth, Kasvi, Brazil). The tubes were incubated at 37 °C for 7 h with shaking at 210 rpm (TE-4200; Tecnal, Piracicaba, Brazil) to enter the stationary phase and reach an *L. monocytogenes* concentration of around 10^9^ CFU/mL. Subsequently, the tubes were centrifuged at 5000 rpm for 5 min, and the supernatant was removed. The resulting pellets were resuspended in 5 mL of 0.1 M phosphate buffer solution (pH 7.2) and homogenized in a vortex (Digital Vortex Mixer, Fisher Scientific, Pittsburgh, PA, USA), forming the *L. monocytogenes* suspension [24].

### 2.3. Mortadella Inoculation

Fifty microliters of the *L. monocytogenes* suspension was distributed onto a slice of mortadella, which was placed on a sterile Petri dish. After an absorption period of 15 min, the samples were subjected to PL treatments described in the following subsection. Samples without the application of PL treatment were used as a control.

### 2.4. Application of Pulsed Light (PL) on Sliced Mortadellas

PL was applied using the benchtop X–1100 System (Xenon Corporation, Wilmington, NC, USA), whose specifications were detailed by [24]. The samples were individually positioned in the chamber’s center at 10.95 cm from the xenon lamp, resulting in 9.92 cm between the lamp and the sample top surface. The applied voltage was 3000 V, and the PL treatment was carried out in a climate-controlled room at 21 °C. Four different PL conditions were selected with variations in pulse width and number of pulses as follows: T_1260_P_2_ = pulse width of 1260 µs and 2 pulses; T_1260_P_3_ = pulse width of 1260 µs and 3 pulses; T_2520_P_1_ = pulse width of 2520 µs and 1 pulse; and T_2520_P_2_ = pulse width of 2520 µs and 2 pulses. These PL parameters were selected based on preliminary tests. Immediately after the pulse was applied, the mortadella slices were vacuum-packed and stored at 4 °C for up to 30 days. The incident fluence (J/cm^2^) on the samples in each pulse was measured with the help of an Ophir Optronics Inc. radiometer, model Nova II, equipped with a pyroelectric sensor model L40 150 A, both from North Logan, UT, USA. This device was positioned in the same spot as the samples (exact distance between the surface and the lamp), and fluence readings were taken in triplicate. The irradiance (W/cm^2^) was calculated using the fluence value divided by the total treatment time [24].

### 2.5. Mortadella Physicochemical Parameters

Temperature, pH, water activity (*a_w_*), and color analyses were performed to characterize sliced mortadella samples before and after the PL treatment. The surface temperature of the slice before and after PL treatment was measured using a digital infrared thermometer (Fluke 62 Max, Fluke Corp, Everett, WA, USA). Twenty slices were used to measure the temperature for each PL treatment condition. Five untreated and five PL-treated slices were used for pH and *a_w_* analyses. pH measurements were taken using a pH meter (Model DM 23, Digimed, São Paulo, Brazil), equipped with a glass electrode probe, in a 1:10 mixture of the sample and distilled water, calibrated with pH 4 and 7 buffer solutions. Water activity (*a_w_*) was determined using an Aqualab 4TE device (Decagon, Pullman, WA, USA).

Instrumental color determination was performed on twenty slices using the Delta Vista 450G colorimeter (Delta Color, São Leopoldo, Brazil) configured with illuminant D65, an observation angle of 10°, and an aperture of 1.5 cm. The same slice was evaluated for L*, a*, and b* values before and after PL treatment. In addition, the ΔE (total color difference) was calculated considering the variations in L*, a*, and b* values after (subscript 2) and before (subscript 1) PL treatment, as in Equation (1).

(1) $$\Delta E={\left[{\left(L_{2}^{*}-L_{1}^{*}\right)}^{2}+{\left(a_{2}^{*}-a_{1}^{*}\right)}^{2}+{\left(b_{2}^{*}-b_{1}^{*}\right)}^{2}\right]}^{0.5}$$

### 2.6. L. monocytogenes Counting

*L. monocytogenes* enumeration in inoculated samples treated and untreated by PL (control) was performed in triplicate. The samples were placed in sterile bags, diluted (1:10) with 15 mL of 0.1% peptone water, and manually massaged for approximately 30 s to extract the bacterial cells, and thus, the first dilution was obtained. Afterward, five serial dilutions (1:10) were made in 0.1% peptone water. Then, 100 µL was surface-plated on 90 mm Petri dishes containing PCA agar (Plate Count Agar, Kasvi) and spread using a disposable sterile Drigalski spatula in a T shape. The plates were incubated inverted at 37 °C for 48 h, and the results were expressed in log CFU/cm^2^ of mortadella. To calculate the logarithmic reduction in the count of *L. monocytogenes* caused by PL application, the log CFU/cm^2^ count in the inoculated samples untreated with PL was subtracted from the bacterial count in inoculated samples treated with PL. Non-inoculated samples without PL treatment were also analyzed to ensure the absence of any bacteria in mortadella samples.

### 2.7. Mortadella Oxidative Stability after Storage

The oxidative stability of non-inoculated samples was evaluated on the 1st and 30th days of storage at 4 °C through TBARS and sensory profile analysis. The four PL treatments and a control sample without PL application were analyzed. The TBARS analysis was performed in triplicate according to the methodology described by Bruna et al. (2001), and the results were expressed in mg of malondialdehyde per kg of sample [26].

The sensory profile was evaluated by 15 trained panelists (9 women and 6 men) over 18 years old in two sessions for each analysis day. The samples were identified with a three-digit code and presented to the panelists monadically and in complete balanced blocks [27]. The panelists evaluated the attributes “pink color” and “oxidized color” of the vacuum-packed mortadella slices, replicating consumers’ perspectives when considering the product in commercial conditions. A 9 cm unstructured scale was used, where the left side corresponded to “little” and the right side to “much”. The sensory tests were conducted in booths under controlled light and temperature conditions. For safety reasons, flavor and aroma attributes related to lipid oxidation were not evaluated, as the PL equipment was located in a Level 2 Biosafety environment.

### 2.8. Statistical Analysis

The experiment was repeated three times. The results of the physicochemical and microbiological analyses were analyzed using a generalized linear model considering “treatments” and “storage time” as fixed effects and repetitions as random effects. The interaction between “treatments” and “storage time” was also analyzed when relevant. The Tukey test (*p* < 0.05) was used to compare means. A GPA (Generalized Procrustes Analysis) map generated from a matrix with 5 rows (5 treatments) and 30 columns (2 descriptors and 15 panelists) was applied for the evaluation of sensory analysis data.

## 3. Results and Discussion

### 3.1. Parameters of PL Applied to the Sliced Mortadellas and the Resulting Fluence and Irradiance

Table 1 illustrates how different pulse width settings and the number of pulses, applying a voltage of 3000 V, influence fluence and irradiance during the treatment of samples. Notably, fluence, which represents the total amount of optical energy (J) delivered per area of the sample, and irradiance, which is the fluence distributed per unit of time (W/cm^2^), are directly affected by the variation in these parameters [24].

As expected, an increase in the number of pulses increased fluence, representing more energy delivered to the sample. However, it is worth highlighting that a fluence increase does not translate into a higher irradiance, which remained approximately constant for treatments with the same pulse width, regardless of the number of pulses. The observed behavior is characteristic of the PL treatment. It indicates that the energy efficiency and effectiveness of the treatment depend not only on the total amount of energy delivered (fluence) but also on how this energy is distributed over time (irradiance).

Interestingly, Table 1 also revealed that treatments with the same pulse width but different numbers of pulses (such as T_2520_P_1_ and T_2520_P_2_) showed that the fluence doubles with twice the number of pulses while irradiance remains relatively stable. As observed, irradiance is a stable device parameter for a given voltage and pulse width, corroborating that energy efficiency and inactivation potential are more closely related to irradiance and treatment duration than to fluence alone [24].

The data analysis further demonstrated that treatments with a longer pulse width (2520 µs) resulted in lower irradiance when compared to those of shorter width (1260 µs), even when the fluence was similar. It indicates that a higher irradiance, applied over a shorter period, may not be as effective as a lower irradiance applied over a more extended period, especially considering the production of reactive oxygen species (ROS) and the subsequent microbial inactivation [28].

Therefore, differentiating between fluence and irradiance is crucial to understanding the efficacy of PL treatments. While fluence indicates the total amount of energy delivered, irradiance determines the rate at which this energy is applied, significantly influencing the treatments’ inactivation results and energy efficiency.

### 3.2. Effect of PL on Temperature, pH, and a_w_ of Sliced Mortadella

**Figure 1:**
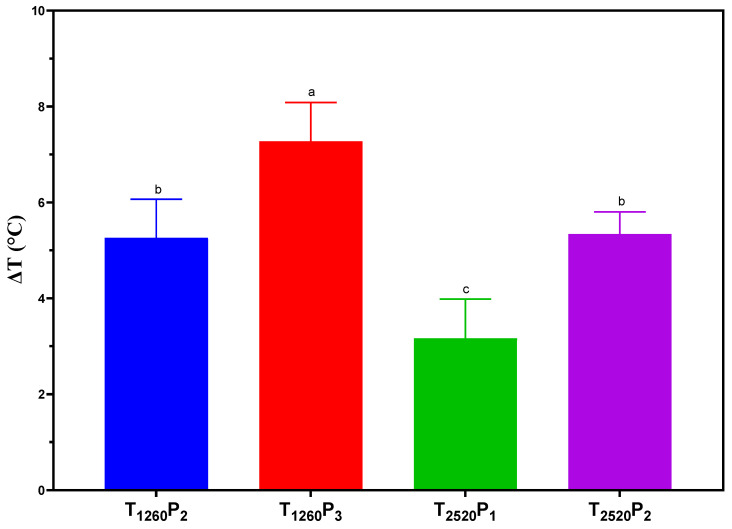
Variation in the temperature on the sliced mortadella surface caused by pulsed light (PL) application. Different letters indicate significant differences based on Tukey’s test (*p* < 0.05). Error bars depict the standard error of the average. Treatments: see Table 1.

PL emits broad-spectrum energy, which can result in sample heating. A maximum variation of around 7 °C caused by PL application was observed, as presented in Figure 1. It was observed that a higher number of pulses provided a greater increase in the temperature of the mortadella slices. This temperature increase occurred due to the delivery of energy in shorter intervals, a characteristic of applying a higher number of pulses. With each additional pulse, there is a rapid injection of energy into the sample, which does not have sufficient time to dissipate between pulses, culminating in a progressive accumulation of heat in the sample [29]. Borges et al. [23] observed a similar phenomenon, reporting that increased pulses raised the temperature of cured and smoked pork loin samples by up to 19.7 °C. Similarly, in their studies on pork meat, Koch et al. [30] also observed a temperature increase ranging from 17.2 to 24.1 °C. These results highlight the importance of controlling the number of pulses in the application of PL, as excessive thermal changes can cause structural and compositional alterations in mortadella, potentially accelerating lipid oxidation and color modifications, which could reduce consumer acceptance.

**Figure 2:**
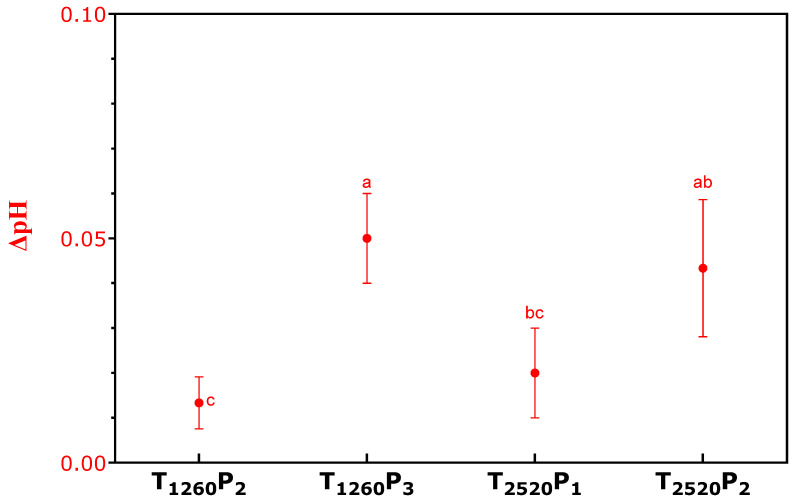
Variations in pH of sliced mortadellas caused by pulsed light (PL) application. Different letters indicate significant differences based on Tukey’s test (*p* < 0.05). Error bars depict the standard error of the average. Treatments: see Table 1.

The variations in pH caused by PL application in the mortadella samples are presented in Figure 2. Interestingly, treatments with the same pulse width but with more pulses showed a more significant increase in pH. This phenomenon can be attributed to several factors. Firstly, the application of PL may have caused the release of basic compounds from the meat proteins, especially under a higher number of pulses, which provides more intense heating [31]. Another possibility is that PL may have reduced the product’s concentration of organic acids or other acidic components, increasing pH [32]. These changes in pH are important, as they can influence the texture, flavor, and microbiological stability of the mortadella, highlighting the need for additional studies to understand these effects better and optimize the application of PL in meat products. It is observed that irradiance does not have a direct relationship with variations in temperature and pH; however, as fluence increases, there is an increase in both temperature and pH.

Simultaneously, applying PL increased the *a_w_* values to less than 0.005. Although this change is technically measurable, it falls within the error range of the equipment used. This result indicates that the application of PL did not have a significant practical impact on the *a_w_* levels.

### 3.3. PL Effect on the Instrumental Color of Sliced Mortadella

**Figure 3:**
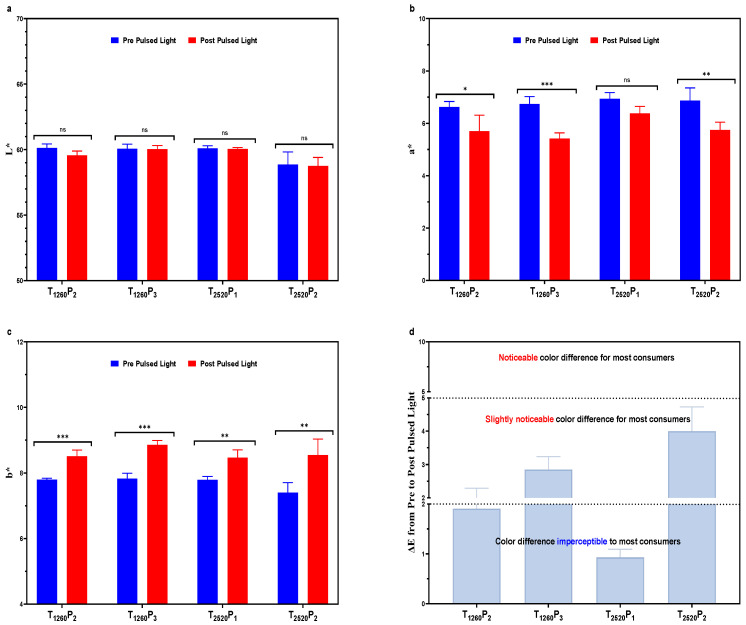
Changes in instrumental color ((**a**): L*; (**b**): a* and (**c**): b*) and ΔE values (**d**) of sliced mortadellas caused by pulsed light (PL) application. Significance: ns (*p* > 0.05); * (*p* < 0.05); ** (*p* < 0.01); *** (*p* < 0.001). Treatments: see Table 1.

The variations in the color of the mortadellas, as objectively measured using instrumental analysis, are presented in Figure 3. It was observed that the different PL conditions applied did not significantly impact the L* values of the mortadellas (Figure 3a). However, there was a significant reduction in the a* values (Figure 3b) and a significant increase in the b* values (Figure 3c) in most of the PL conditions tested. The exception was the T_2520_P_1_ treatment, which used only one pulse and did not cause a significant reduction in the a* values. The most significant effect (*p* < 0.001) was observed in the T_1260_P_3_ treatment, which had the highest number of pulses and the highest temperature increase (Figure 1). These changes in the instrumental color of the mortadellas can be attributed to different aspects of PL. For example, the decrease in red intensity, indicated by the lower a* values, may have been caused by the denaturation of nitrosylhemochrome, both due to the heat locally generated and the exposure to ultraviolet and visible light from PL [30,33]. Regarding the increase in yellowish hue, indicated by the rise in b* values, a possible explanation is that the energy from PL generates surface heat and accelerates lipid oxidation. This oxidation can form compounds that alter fats’ appearance, giving a yellowish tonality to food [34].

The ΔE, which represents the total color difference, was calculated for each treatment condition with PL to assess whether the modifications in L*, a*, and b* values resulted in sensorily perceptible changes in the color of the mortadella (Figure 3d). The results showed that the treatment with only one pulse (T_2520_P_1_) resulted in the lowest ΔE value, less than 1, followed by the treatment with two pulses and a pulse width of 1260 μs (T_1260_P_2_), which had a ΔE less than 2. ΔE values below 2 are generally considered almost imperceptible by most consumers [35], indicating that these PL conditions caused minimal changes in the color of the mortadella.

On the other hand, treatments with three pulses and 1260 μs (T_1260_P_3_) and with two pulses and 2520 μs (T_2520_P_2_) had ΔE values in the range of 2 to 5, which are slightly perceptible and perceptible, respectively, for most consumers [35]. These treatments had higher fluence, 6.57 and 5.28 J/cm^2^, respectively, in contrast to those with lower ΔE, which had a fluence of 2.64 and 4.28 J/cm^2^. These results suggest, therefore, that both the number of pulses and the higher fluence may be correlated with the most significant modifications in the color of the mortadella.

### 3.4. PL Effect on the Reduction in L. monocytogenes

**Figure 4:**
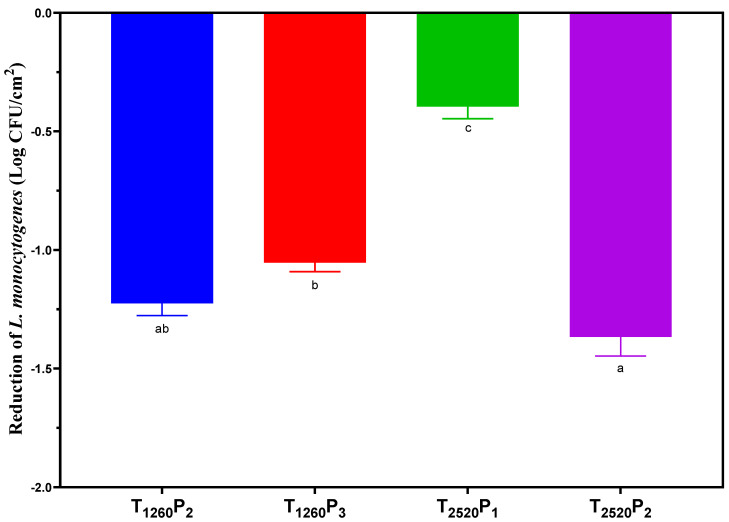
Reduction in *L. monocytogenes* (Log CFU/cm^2^) of sliced mortadellas caused by pulsed light (PL) application. Different letters indicate significant differences based on Tukey’s test (*p* < 0.05). Error bars depict the standard error of the average. Treatments: see Table 1.

The logarithmic reduction in *L. monocytogenes* caused by PL application is presented in Figure 4. The PL conditions tested resulted in a reduction ranging between 0.43 and 1.44 Log CFU/cm^2^, indicating the efficacy of PL in decreasing the microbial load. When treatments with lower fluence were compared, it was observed that T_1260_P_2_ (4.38 J/cm^2^) achieved a significantly higher reduction (*p* < 0.05) than T_2520_P_1_ (2.64 J/cm^2^). This greater reduction in T_1260_P_2_ can be attributed to the higher number of pulses and a greater irradiance, which likely intensified the exposure to PL energy, causing more effective damage to the cellular structure and DNA of the bacteria [36,37].

When treatments with a pulse width of 1260 μs were compared, a similar reduction in *L. monocytogenes* (*p* > 0.05) was found, even with differences in fluences (4.38 and 6.57 J/cm^2^) and the number of pulses (two and three). This similarity suggests that, in this specific range, variations in the fluence and number of pulses may not have been sufficient to create a significant difference in microbial reduction efficacy. In addition, T_1260_P_2_ and T_1260_P_3_ treatments have exhibited similar high irradiances (1738.3 ± 5.2 W/cm^2^ and 1738.8 ± 4.8 W/cm^2^, respectively). This trend aligns with the findings of Xie et al. [38], who reported that while pulse width and irradiance are constant, the number of pulses does not significantly affect inactivation efficiency.

In contrast, the results of treatments with a pulse width of 2520 μs revealed that the T_2520_P_2_ treatment, with two pulses and a fluence of 5.28 J/cm^2^, had a significantly higher reduction (*p* < 0.05) of approximately 1 log CFU/cm^2^ compared to T_2520_P_1_, which had one pulse and a fluence of 2.64 J/cm^2^. This result may be related to the higher number of pulses and the higher fluence applied. Conversely, treatments T_2520_P_1_ and T_2520_P_2_, with a longer pulse width of 2520 µs, showed lower irradiances (1049.3 ± 3.6 W/cm^2^ and 1046.9 ± 3.5 W/cm^2^, respectively). This result reflects Xie et al.’s [38] finding of a negative relationship between inactivation efficiency and pulse number at lower irradiance.

Interestingly, T_2520_P_2_, with a performance similar to T_1260_P_2_, suggests that the ideal combination of pulse width, number of pulses, fluence, and irradiance is crucial for maximizing efficacy against *L. monocytogenes*, highlighting the complexity and non-linear nature of the relationship between PL treatment parameters and microbial reduction efficacy.

The current literature offers limited studies focusing on the inactivation of *L. monocytogenes* by PL in sliced mortadella. The reduction achieved in this study slightly exceeded the outcomes reported by Hierro et al. [39], who documented a 1.11 Log CFU/cm^2^ reduction for *L. monocytogenes* with the application of PL (2–3 pulses, 250 µs per pulse and 8.4 J/cm^2^) in vacuum-packaged mortadella slices. This result is consistent with earlier findings by Fernández et al. [40] which demonstrated PL’s capability to penetrate packaging films of varying compositions and thicknesses.

The efficacy of PL may also have been influenced by the mortadella matrix, characterized by irregularities and roughness, which may have created a shading effect or even protection against UV light, especially considering the thick polysaccharide cell layer of *L. monocytogenes*, which can protect the cell nucleus and its DNA from lethal light discharges [11]. In this sense, Hierro et al. [39] reported a higher *L. monocytogenes* inactivation on cooked ham than in mortadella, demonstrating that the surface topography greatly impacts the antimicrobial effect of PL. Results from other studies corroborate this variation in PL efficacy depending on the food matrix. Borges et al. [23] observed a reduction of up to 1.58 Log CFU/g in *L. monocytogenes* in cured loin using a fluence of 5.31 J/cm^2^. Similarly, Fernández et al. [41] reduced *L. innocua* by 2 Log CFU/cm^2^ on the surface of dry-cured ham with a fluence of 8.4 J/cm^2^. These studies show that differences in pathogen reduction after PL application can be attributed to the characteristics of the matrix, such as fat content and surface homogeneity, suggesting that smoother and more homogeneous surfaces allow for greater pathogen reduction. In addition to the matrix variability, strain-by-strain differences in *L. monocytogenes* resistance may also contribute to variability in reduction rates across studies. Different strains may exhibit distinct resistance levels to PL treatment due to their genetic and structural differences, which can affect their vulnerability to UV light and the overall efficacy of microbial inactivation. Therefore, these results highlight the importance of considering the specific characteristics of the food matrix and the biological properties of the pathogen when evaluating the efficacy of PL. The fact that PL resulted in a reduction of up to 1.44 Log CFU/cm^2^ in this study is notable, considering the robust characteristics of *L. monocytogenes* and the irregularities of this meat product.

### 3.5. PL Effect on the Oxidative Stability of Sliced Mortadella

**Figure 5:**
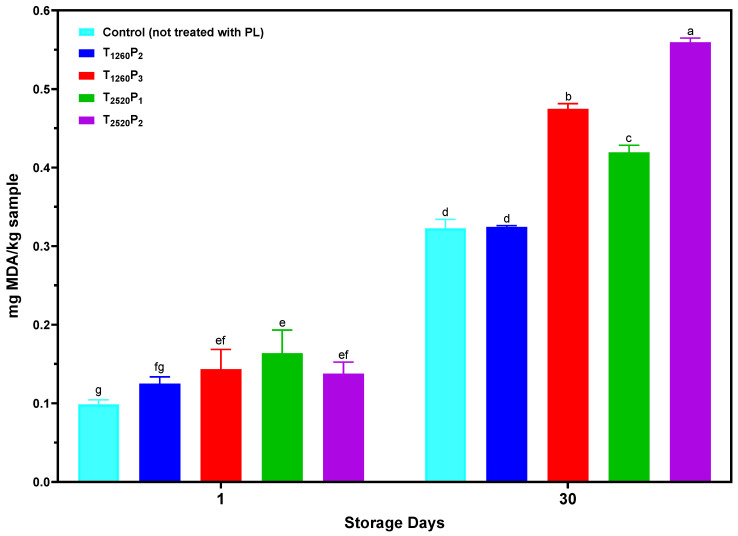
TBARS values of sliced mortadellas during storage. Different letters indicate significant differences based on Tukey’s test (*p* < 0.05). Error bars depict the standard error of the average. Treatments: see Table 1.

The results of the TBARS analysis of mortadella samples during storage are presented in Figure 5. A significant interaction between treatments and storage time was observed (*p* < 0.05). The application of PL in almost all tested conditions, except for T_1260_P_2_, increased TBARS values compared to the control (not treated with PL). This increase in TBARS values after PL application can be attributed to the intense luminous energy potentially accelerating lipid oxidation reactions [42]. Similar studies also reported increased lipid oxidation shortly after PL application in different meat products [32,33,43].

After 30 days of storage, an increase in TBARS values was observed in all treatments, including the control. Notably, the highest values were found in treatments with higher fluences (T_2520_P_2_: 5.28 J/cm^2^; T_1260_P_3_: 6.57 J/cm^2^). A plausible explanation is that a higher fluence provides more energy [42] and increases the speed of oxidative reactions, leading to a higher TBARS index. On the other hand, the T_1260_P_2_ treatment, with a fluence of 4.38 J/cm^2^, showed TBARS values similar (*p* > 0.05) to the control after 30 days. This result suggests that applying a lower fluence and a greater irradiance may effectively mitigate the undesirable effects of PL on the oxidative stability of mortadella. Additionally, the observation that T_2520_P_1_ showed higher TBARS values on the 30th day of storage than T_1260_P_2_ highlights the importance of pulse width as a critical factor, mainly as T_2520_P_1_ had the lowest fluence of all treatments. Therefore, this variable should also be carefully considered and adjusted to optimize the effects of PL and preserve product quality over time.

**Figure 6:**
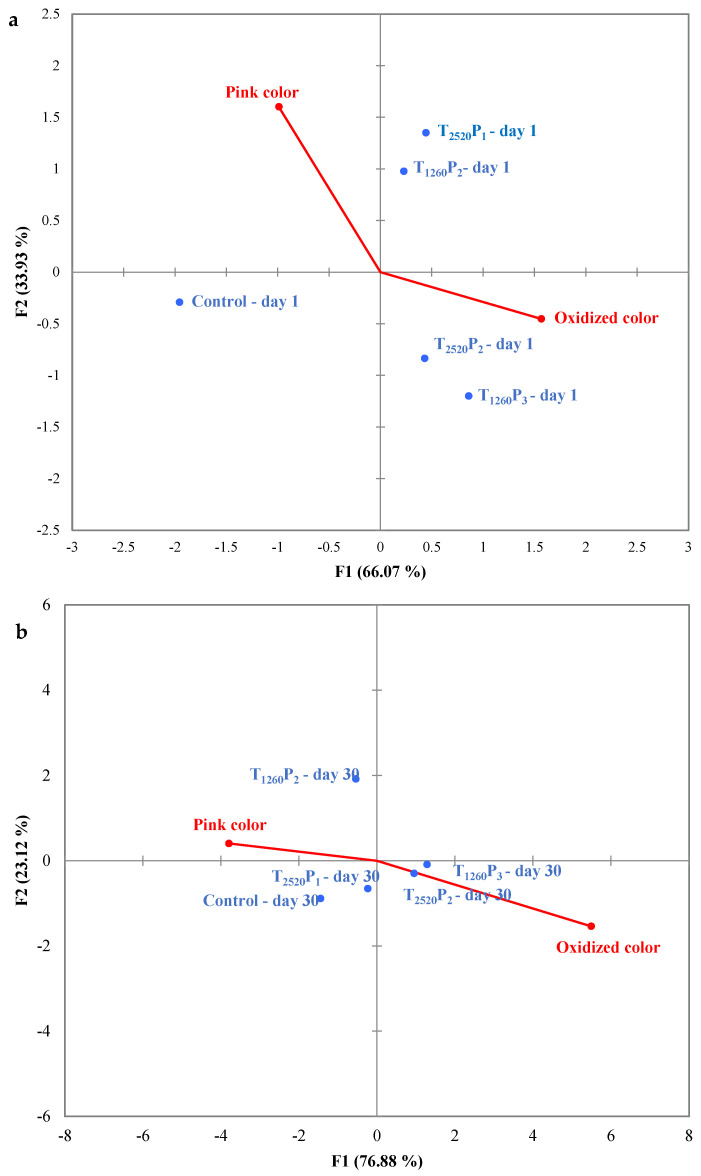
Generalized Procrustes Analysis (GPA) map for sensory analysis results of sliced mortadellas on day 1 (**a**) and day 30 (**b**) of storage. Treatments: see Table 1.

The GPA map used to evaluate the sensory analysis results of mortadella samples is presented in Figure 6. Interestingly, samples with fluences of 6.57 and 5.28 J/cm^2^ (T_1260_P_3_ and T_2520_P_2_, respectively) were characterized by the attribute “oxidized color”, both at the beginning (Figure 6a) and at the end (Figure 6b) of the storage period. This observation is consistent with the highest values of ΔE (Figure 3) and TBARS (Figure 5) found in these samples. On the other hand, samples with a fluence of 4.38 and 2.64 J/cm^2^ (T_1260_P_2_ and T_2520_P_1_) were positioned close to the control, especially on the 30th day of storage, and were characterized by the attribute “pink color”. This result is particularly interesting as it indicates that, despite the modifications in color and TBARS values caused by PL, these changes were not pronounced enough to be perceived by consumers.

## 4. Conclusions

This study demonstrated the potential of applying pulsed light (PL) to sliced mortadella, analyzing how parameters such as pulse width (1260 to 2520 µs), number of pulses (one to three), fluence (2.64 to 6.57 J/cm^2^), and irradiance (1046.9 to 1738.3 W/cm^2^) affect color, oxidative stability, and *L. monocytogenes* count. The T_1260_P_2_ treatment (1260 µs pulse width, two pulses, 4.38 J/cm^2^ fluence, and 1738.8 W/cm^2^ irradiance) provided an effective balance between pathogen reduction and maintaining color and oxidative stability. The 1.44 log CFU/cm^2^ reduction in *L. monocytogenes* indicates a promising strategy for enhancing the safety of ready-to-eat products. Future research should explore other variables such as packaging types, storage conditions, and the influence on other pathogens and quality characteristics to further optimize this technology for sliced meat products.

## Figures and Tables

**Table 1:** Parameters of pulsed light applied to the sliced mortadellas and the resulting fluence and irradiance.

Treatments	Pulse Width (µs)	Number of Pulses	Fluence (J/cm^2^)	Irradiance (W/cm^2^)
T_1260_P_2_	1260	2	4.38 ± 0.01 ^c^	1738.3 ± 5.2 ^a^
T_1260_P_3_	1260	3	6.57 ± 0.01 ^a^	1738.8 ± 4.8 ^a^
T_2520_P_1_	2520	1	2.64 ± 0.01 ^d^	1049.3 ± 3.6 ^b^
T_2520_P_2_	2520	2	5.28 ± 0.01 ^b^	1046.9 ± 3.5 ^b^

Mean values ± standard deviation (*n* = 3 replicates). Different letters indicate significant differences based on Tukey’s test (*p* < 0.05).

## Data Availability

The original contributions presented in the study are included in the article, further inquiries can be directed to the corresponding author.
